# Concentration-response gene expression analysis in zebrafish reveals phenotypically-anchored transcriptional responses to retene

**DOI:** 10.3389/ftox.2022.950503

**Published:** 2022-08-25

**Authors:** Lindsay B. Wilson, Ryan S. McClure, Katrina M. Waters, Michael T. Simonich, Robyn L. Tanguay

**Affiliations:** ^1^ Department of Environmental and Molecular Toxicology, Sinnhuber Aquatic Research Laboratory, Oregon State University, Corvallis, OR, United States; ^2^ Biological Sciences Division, Pacific Northwest National Laboratory, Richland, WA, United States

**Keywords:** zebrafish, benchmark concentration, phenotypic anchoring, retene, toxicogenomics, polycyclic aromatic hydrocarbon (PAH), aryl hydrocarbon receptor

## Abstract

Polycyclic aromatic hydrocarbons (PAHs) are ubiquitous environmental contaminants and are associated with human disease. Canonically, many PAHs induce toxicity via activation of the aryl hydrocarbon receptor (AHR) pathway. While the interaction between PAHs and the AHR is well-established, understanding which AHR-regulated transcriptional effects directly result in observable phenotypes and which are adaptive or benign is important to better understand PAH toxicity. Retene is a frequently detected PAH in environmental sampling and has been associated with AHR2-dependent developmental toxicity in zebrafish, though its mechanism of toxicity has not been fully elucidated. To interrogate transcriptional changes causally associated with retene toxicity, we conducted whole-animal RNA sequencing at 48 h post-fertilization after exposure to eight retene concentrations. We aimed to identify the most sensitive transcriptomic responses and to determine whether this approach could uncover gene sets uniquely differentially expressed at concentrations which induce a phenotype. We identified a concentration-response relationship for differential gene expression in both number of differentially expressed genes (DEGs) and magnitude of expression change. Elevated expression of *cyp1a* at retene concentrations below the threshold for teratogenicity suggested that while *cyp1a* expression is a sensitive biomarker of AHR activation, it may be too sensitive to serve as a biomarker of teratogenicity. Genes differentially expressed at only non-teratogenic concentrations were enriched for transforming growth factor-β (TGF-β) signaling pathway disruption while DEGs identified at only teratogenic concentrations were significantly enriched for response to xenobiotic stimulus and reduction-oxidation reaction activity. DEGs which spanned both non-teratogenic and teratogenic concentrations showed similar disrupted biological processes to those unique to teratogenic concentrations, indicating these processes were disrupted at low exposure concentrations. Gene co-expression network analysis identified several gene modules, including those associated with PAHs and AHR2 activation. One, Module 7, was strongly enriched for AHR2-associated genes and contained the strongest responses to retene. Benchmark concentration (BMC) of Module seven genes identified a median BMC of 7.5 µM, nearly the highest retene concentration with no associated teratogenicity, supporting the hypothesis that Module seven genes are largely responsible for retene toxicity.

## 1 Introduction

High-throughput screening (HTS) of chemicals and elucidation of molecular mechanisms of toxicity often rely on *in vitro* methods (Tice et al., 2013). *In vitro* models, while efficient and useful for tissue-specific effects, cannot holistically assess chemical interactions to the degree *in vivo* models can, with complex metabolism and fully integrated tissues. The early life-stage zebrafish (*Danio rerio*) is a popular model for HTS of chemicals to detect those which have biological impacts. Zebrafish develop externally and rapidly with transparent tissues, allowing for visual observation of teratogenic effects by 5 days post-fertilization (Kimmel et al., 1995). Additionally, zebrafish share high genetic homology with mammals and possess many of the same organ systems, making it an excellent model for transcriptomic assessment of chemical effects translatable to human health (Howe et al., 2013).

Zebrafish have been utilized to identify complex chemical-transcriptome interactions and identify differentially expressed genes (DEGs) important to overall toxicity response (Huang et al., 2014; Huang et al., 2015; Souza et al., 2015; Shankar et al., 2019). One major approach is through RNA sequencing, which provides an unbiased snapshot of all gene expression changes at a given timepoint during development in response to chemical insult (Heijne et al., 2005). Many transcriptomic studies have revealed important gene expression changes in response to polycyclic aromatic hydrocarbons (PAHs) across model systems (Song et al., 2012; Jayasundara et al., 2015; Brinkmann et al., 2016; Shankar et al., 2019). PAHs are ubiquitous environmental contaminants produced both naturally and anthropogenically through sources like wildfires, crude oil, and cigarette smoke. PAHs have been associated with neurobehavioral and teratogenic effects in laboratory studies and increased risk of cancers in humans (Boffetta et al., 1997; Billiard et al., 1999; Bostrom et al., 2002; Armstrong et al., 2004; Knecht et al., 2013; Knecht et al., 2017; Geier et al., 2018a; Geier et al., 2018b). Mechanistically, many PAHs initiate toxic responses through activation of the aryl hydrocarbon receptor (AHR) pathway. The expression of one of the genes in its regulon, *cyp1a,* is the primary biomarker of AHR activation and exposure to PAHs. However, while elevated *cyp1a* often indicates AHR activation, it does not automatically mean a chemically-induced phenotype will develop *in vivo* (Hu et al., 2007).

Teratogenic phenotypes resulting from PAH exposure are fundamentally based on changes in gene expression patterns. RNA sequencing is an efficient method with which to uncover those pattern changes and begin to identify the mechanisms of toxicity. With a sufficient amount of data, a gene expression network can be inferred to reveal dysregulated pathways related to individual chemicals or chemical classes, can highlight which processes may be linked within a biological system, can identify genes that may respond in a more subtle manner and can incorporate centrality measures to identify which genes might be central to a system based on co-expression values with other genes (Mcdermott et al., 2009; Pohl et al., 2021; Shankar et al., 2021). A 2021 study from our research group compiled phenotypically-anchored RNA sequencing data from 48 h post-fertilization (hpf) zebrafish developmentally exposed to 10 flame retardant chemicals, 22 PAHs, and 2,3,7,8-Tetrachlorodibenzodioxin (TCDD), a strong AHR agonist commonly used to investigate AHR-regulated effects (Shankar et al., 2021). We inferred a gene co-expression network and identified chemical-specific gene modules, including one, Module 13, which contained highly-connected genes definitively belonging to the AHR2 signaling pathway. AHR2 is the zebrafish ortholog most functionally similar to the human AHR.

In zebrafish, gene expression changes at 48 hpf have been linked to observed toxicity at 5 days post-fertilization with chemical exposure at non-teratogenic concentrations producing few dysregulated genes (Rericha et al., 2022). Whether toxic concentrations of AHR activators produce different DEGs compared to non-toxic concentrations or similar DEGs but to a different degree is not well understood. While we have a good understanding of what gene expression changes are induced by AHR activation, which of these transcriptional changes are directly associated with toxicity and which are adaptive or benign is not possible to deduce from assessment using one exposure concentration. Retene, a frequently detected PAH in environmental sampling, induces elevated *cyp1a* expression in developing zebrafish in an AHR2-dependent manner and produces malformations similar to those induced by TCDD (Geier et al., 2018b; Shankar et al., 2019). In this study, we leveraged an established developmental zebrafish screening assay and whole-animal transcriptomic analysis to identify concentration-dependent gene expression changes associated with retene teratogenicity. We aimed to identify the most sensitive transcriptomic responses and to determine whether this approach could uncover gene sets uniquely differentially expressed at concentrations which induce a phenotype.

## 2 Materials and methods

### 2.1 Zebrafish methods

#### 2.1.1 Husbandry and exposures

Specific pathogen-free wild type 5D zebrafish (*Danio rerio*) and an AHR2 mutant fish line (ahr2hu3335) (Goodale et al., 2012) were reared at the Sinnhuber Aquatic Research Laboratory (SARL) in accordance with Institutional Animal Care and Use Committee protocols at Oregon State University (IACUC-2021-0166 and 2021-0227) (Kent et al., 2011). Fish were housed on a recirculating water system kept at 28° ± 1 C under a 14 h light: 10 h dark cycle in 50- or 100-gallon brood stock tanks. Water was supplemented with Instant Ocean salts (Spectrum Brands, Blacksburg, VA, United States ) and sodium bicarbonate to maintain pH 7.4. Fish were fed with Gemma Micro twice daily (Skretting, Inc., Fontaine Les Vervins, France) (Barton et al., 2016).

Embryos were collected from adult fish using an internal embryo collection apparatus on the day of exposure, sorted by developmental stage, and kept in E2 embryo medium (EM) consisting of 15 mM NaCl, 0.5 mM KCl, 1 mM CaCl2, 1 mM MgSO4, 0.15 mM KH2PO4, 0.05 mM Na2HPO4, and 0.7 mM NaHCO3 buffered with 1 M NaOH to pH 7.2 in a temperature-controlled incubator at 28 ± 1°C until dechorionation (Westerfield, 2000). At 4 h post-fertilization (hpf), embryos were enzymatically dechorionated using a custom-made apparatus previously described (Mandrell et al., 2012). Following dechorionation, embryos were screened for enzymatic or mechanical damage under a dissecting microscope and robotically loaded into 96-well plates prefilled with 100 μL EM.

Retene (CAS 483-65-8, Santa Cruz Biotechnology, 97% purity) and 100% dry dimethyl sulfoxide (DMSO) were dispensed into 96-well plates pre-loaded with six hpf embryos and 100 μL EM using an HP D300 or D300e Digital Dispenser then immediately sealed using an Eppendorf 5390 heat sealer with pressure-sensitive silicone adhesive backed polyolefin plastic PCR film (Thermaseal RTS). Plates were incubated overnight at 28° ± 1°C on an orbital shaker at 235 RPM under dark conditions. Embryos were exposed to initial range-finding nominal water concentrations of 0, 1, 2.54, 6.45, 16.4, 35, 74.8, and 100 µM retene (1% DMSO by volume) beginning at six hpf (1 test plate, n = 12 for each concentration). Definitive testing concentrations were then selected to capture 0%–100% teratogenicity and to assess AHR2-dependence: 0, 1, 5, 20, 35, 50, 65, and 100 µM. For definitive testing, n = 36 fish per concentration across three replicate plates for wild type fish and n = 24 fish per concentration across two replicate plates for ahr2hu3335 fish due to fecundity limitations. All retene exposures were administered exactly as above.

#### 2.1.2 Toxicity screening

To assess developmental toxicity, zebrafish were visually screened for a total of 13 morphological endpoints at 24 and 120 hpf. Table 1 lists morphological endpoints assessed at each timepoint. The percent incidence of abnormalities for each endpoint was calculated across replicate plates. The percent incidence of any observed morphological effect including mortality was calculated and reported here as “any effect”. Images of all measured endpoints can be found at https://github.com/Tanguay-Lab/Bioinformatic_and_Toxicological_Resources/tree/main/Files/Zebrafish_Phenotype_Atlas (accessed 21 March 2022). See Supplementary Table S1 for supporting information regarding binning of morphological endpoints. Figures were generated using R and GraphPad Prism.

**TABLE 1:** All zebrafish morphological endpoints, including mortality, measured at 24 and 120 h post-fertilization (hpf).

Zebrafish Morphological Endpoints	
24 hpf	mortality, delayed progression, spontaneous movement
120 hpf	mortality, edemas, bent axis, touch response, and craniofacial, muscular/cardiovascular, lower trunk, brain, skin, notochord malformations

### 2.2 Transcriptomics

#### 2.2.1 Concentration selection

Following initial developmental toxicity screening, concentrations for RNA-sequencing were selected to cover the range of observed effects with a top concentration of 50 μM at which 100% of animals exhibited malformations or mortality at 120 hpf. Concentrations were then scaled down by a factor of 0.4 for a concentration list of: 0, 0.205, 0.512, 1.28, 3.2, 8, 20, and 50 μM, with a skew toward low concentrations which do not induce teratogenicity.

#### 2.2.2 RNA-seq

At 48 hpf, fish were briefly screened for malformations to ensure only morphologically normal fish were used for transcriptomics. No significant teratogenicity was observed across concentrations. Fish were collected in four pools of nine fish each for RNA sequencing. Pooled fish were briefly anesthetized on ice and excess water was removed. Tissue was immediately homogenized in 1.5 ml Safe-Lock microcentrifuge tubes (Eppendorf, CT, United States ) with 200 µL RNAzol RT (Molecular Research Center) and 0.5 mm zirconium oxide beads in a Bullet Blender tissue homogenizer (Next Advance, NY, United States ) for 3 min, speed 8. An additional 300 µL RNAzol RT was added to each tube and the homogenate was vortexed and kept at -80°C until RNA isolation. Total RNA was isolated using a Direct-zol RNA MiniPrep Kit (Zymo Research, CA, United States ). RNA samples were sent to the Beijing Genomics Institute (BGI) for library preparation and sequencing. RNA integrity was assessed (RIN score > 8.5) using an Agilent Technologies 2100 Bioanalyzer. Briefly, mRNA was purified using oligo (dT)-attached magnetic beads and fragmented. cDNA was synthesized using random hexamer-primed reverse transcription, end-repaired and 3′ adenylated. Adapters were ligated to 3′ adenylated ends and cDNA was PCR amplified and purified. The resulting library was validated using an Agilent Technologies 2100 Bioanalyzer. 100 bp paired-end mRNA sequencing was conducted using BGI’s DNBseq™ platform (DNBSEQ-G400).

#### 2.2.3 Differential gene expression analysis

All fastq files were aligned to the Z11 zebrafish genome (GCF_000002035.6_GRCz11_genomic.fna) using the Star Aligner (Dobin et al., 2013). Resulting SAM files were then used to count reads aligning to genes using HTSeq (Anders et al., 2015) along with the gff file for the Z11 reference genome (GCF_000002035.6_GRCz11_genomic.gff) with default settings and an alignment value cutoff of a10. This led to a file of 39701 genes with raw counts assigned to them. From this file any gene that had a count of ‘0’ in at least 8/32 (25%) conditions was removed from further analysis. This was done so that resulting networks would be of smaller size and easier to work with and because such genes were of very low abundance. This led to a dataset of 22,686 genes across 32 conditions. Raw counts were normalized with DESeq2 for downstream analyses (Love et al., 2014). DESeq2 was also used to identify differentially expressed genes (DEGs) defined as genes with an adjusted *p*-value less than 0.05. ZFIN ids in the gff file were then replaced with gene names using g:Profiler (Raudvere et al., 2019). If the gene had no gene name then the GeneID was retained as a unique identifier. In a few cases (94/22,686 genes), the gene names were identical except for capitalization. These gene were aggregated and the expression values were averaged.

#### 2.2.4 Benchmark concentration modeling

Concentration-response modeling of genes and gene sets was performed and visualized using BMDExpress2 (Yang et al., 2007). Briefly, a two-sided Williams Trend test was performed for each gene across concentrations to identify genes with significant (*p* < 0.05) monotonic response in expression, regardless of directionality. Individual genes which met this criterion were fit to 10 continuous parametric models and the model with the lowest Akaike information criterion (AIC) was selected for benchmark concentration modeling. Benchmark concentrations for gene and gene set-level responses were calculated based on EPA guidance for continuous data with a benchmark response (BMR) factor of 1.349, equivalent to a 10% change from control. Additional parameters included assumed constant variance, 250 maximum iterations and confidence level 0.95. The Hill model was flagged if the model k parameter was below one-third the lowest positive dose. In cases where the Hill model was the best fit, but was flagged for this reason, the next best model with a goodness of fit *p* > 0.05 was selected. Genes with a BMC greater than the highest concentration tested were removed from the analysis. For gene set analysis, the median BMC was used.

#### 2.2.5 Gene co-expression analysis

Gene co-expression networks were inferred using fold change values for genes. Fold change values were calculated for each replicate individually including replicates of the control conditions by calculating an average for each set of control replicates and using that as a basis for determining fold changes. In addition, to reduce overall sizes of the network for ease of analysis, the top 8,000 genes were used based on coefficient of variation (CV). Selecting genes for network inference with a high CV value ensured that genes that had strong changes in expression across samples, those that could be linked to other genes based on expression similarity, would be included in the network inference. To infer networks the random forest algorithm GENIE3 was used (Huynh-Thu et al., 2010). Any co-expression value between gene pairs with an edge weight above 0.0086 was included as an edge in the network. This cutoff led to a network of 4,678 edges (top 0.007% of possible edges) so only the most strongly connected genes were included in the analyzed network. Networks were viewed and modified with colors/node sizes, etc. in Cytoscape (Shannon et al., 2003). The network analyzer app in Cytoscape was also used to identify centrality values of degree and betweenness. To identify modules the fastgreedy algorithm in the R package igraph was used (Csardi and Nepusz, 2006).

An analysis of how edge weights shifted as a function of loss of data specific to each concentration was performed for one module, Module 7. All possible edge weights (those above and below our 0.0086 cutoff for the full network) between two Module seven genes were assessed for a total of 31,506 edges. For each edge, the ratio of the edge weight in the network inferred from the complete dataset was compared to a network inferred after removing data from a single concentration of retene. This process was iteratively performed, removing one concentration from the network at a time and calculating all edge weight ratios compared to the full data network. A ratio of greater than one indicates the edge weight was higher in the control network than a network with the specific concentration dataset removed (i.e. that the co-expression between these two genes was negatively affected by loss of data representing that retene concentration).

## 3 Results

### 3.1 Retene bioactivity screening

**FIGURE 1:**
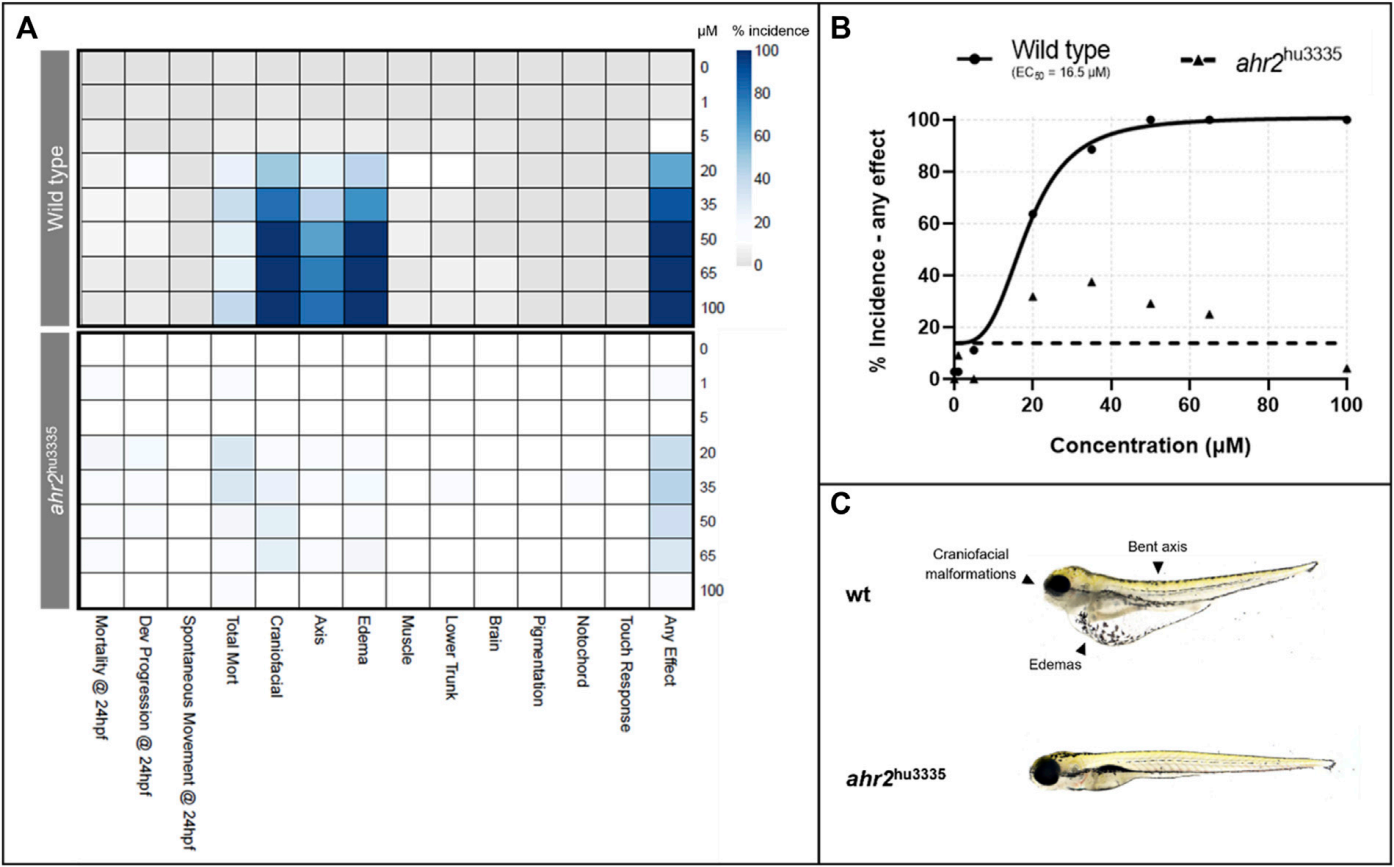
Effects of retene exposure on zebrafish development at 24 and 120 hpf in wild type and AHR2 mutant fish. **(A)** heatmap of endpoint incidence. Depth of color indicates percent incidence (*x*-axis) at given concentration (*y*-axis); **(B)** concentration-response curves for percent incidence of teratogenicity at 24 or 120 hpf; **(C)** 120 hpf larvae resulting from exposure to 50 µM retene beginning at six hpf, with and without functional AHR2.

Wild type and AHR2 mutant (*ahr2*hu3335) fish were exposed to a range of retene concentrations from 0 to 100 µM for initial bioactivity screening and to assess phenotype dependency on AHR2. Initial exposure to retene induced significant craniofacial defects, edemas, bent body axis, and mortality in wild type fish (Figure 1A). Based on the incidence of ‘any effect’ (incidence at any teratogenic endpoint), the concentration resulting in malformations in 50% of wild type animals (EC_50_) was 16.5 µM with 100% of animals exhibiting mortality or teratogenicity at 50 µM (Figure 1B). AHR2 mutant fish showed significantly reduced retene teratogenicity, indicating that the retene toxicity mechanism is AHR2-dependent (Figure 1A–C).

**FIGURE 2:**
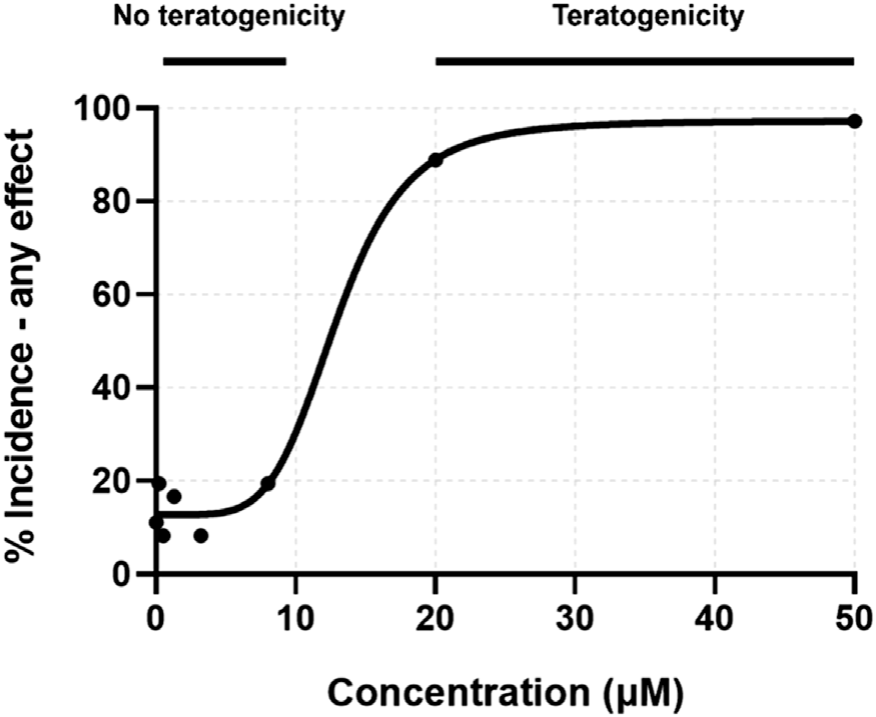
Concentration-response curve for percent incidence of any teratogenic effect observed at 24 or 120 hpf. Exposure concentrations for toxicity screening were matched to those for transcriptomic assessment.

From the initial screening, concentrations were identified which induced 0–100% teratogenicity. Eight concentrations, with a skew toward non-teratogenicity-inducing concentrations, were selected for mRNA sequencing. These concentrations recapitulated the lack of teratogenicity below 20 µM (Figure 2). Percent incidence of developmental toxicity endpoints are found in Supplementary Table S2.

### 3.2 Retene-induced transcriptomic changes

Global gene expression was assessed in 48 hpf zebrafish larvae in response to eight retene concentrations anchored to teratogenicity screening results. Differential expression analysis and benchmark concentration modeling were used to assess concentration-response relationships of individual retene-responsive genes and identify thresholds at which expression of these genes was altered. A gene co-expression network was inferred for genes across all conditions to identify sets of genes most responsive to retene.

#### 3.2.1 Differential gene expression

**FIGURE 3:**
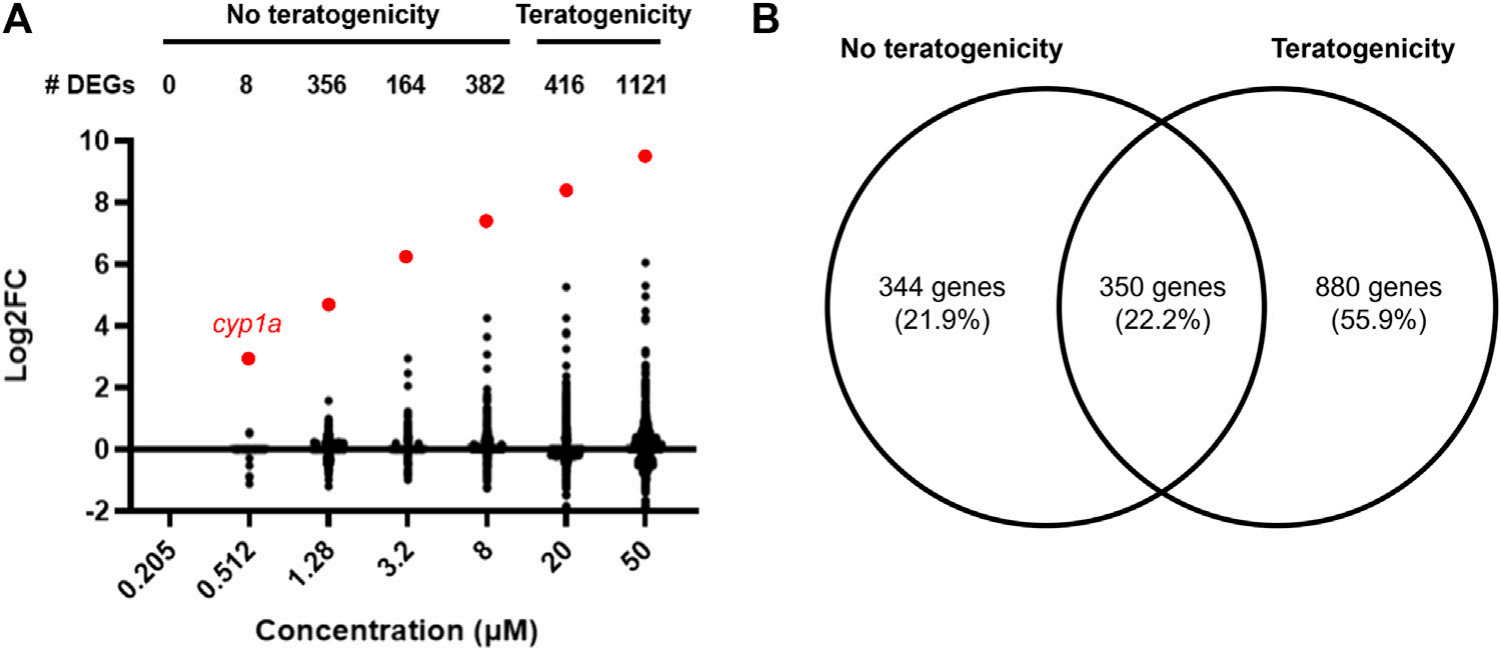
**(A)** Number of differentially expressed genes and their corresponding Log2 fold change values at each retene concentration (adj. *p* < 0.05). *Cyp1a* is highlighted red. **(B)** Venn diagram of DEGs at concentrations with and without teratogenicity at 120 hpf. Overlapping region represents genes which were differentially expressed in at least one non-teratogenic and one teratogenic concentration.

Differentially expressed genes across each concentration were identified (no fold change cut off) with an adjusted *p*-value ≤ 0.05. The number of DEGs generally increased with increasing concentration, with no DEGs identified at 0.205 µM and 8, 356, 164, 382, 416, and 1121 DEGs at 0.512, 1.28, 3.2, 8, 20, and 50 μM, respectively (Figure 3A). Most DEGs had low (<1) log2FC values.

**FIGURE 4:**
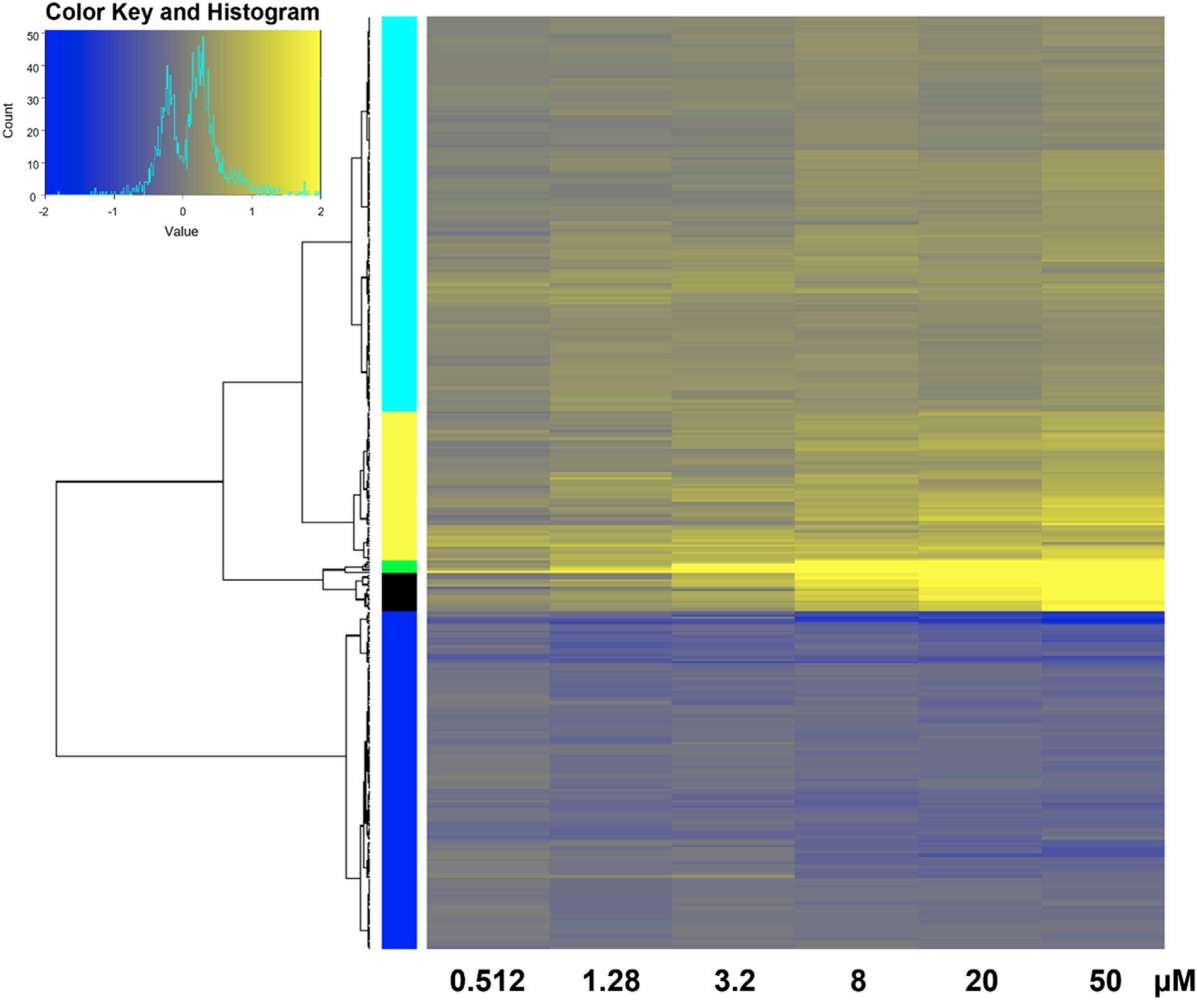
Heatmap of transcriptomic response at each retene concentration for genes common to both non-teratogenic and teratogenic concentrations. Average Log2FC values for each gene at each treatment are displayed with yellow indicating higher expression and blue indicating lower expression compared to DMSO vehicle control. Genes are grouped by ward clustering with gene clusters on the left.

DEG lists were compiled for concentrations which did not induce teratogenicity (0.205–8 µM) versus those which did (20–50 µM) (Figure 3B). 880 DEGs were identified at only teratogenic concentrations indicating these may play an important role in the emergence of teratogenicity. Functional enrichment analysis of these genes using Gene Ontology (GO) terms identified significant (*p* < 0.05) GO terms related to response to toxic substance and reduction and oxidation reactions. Figure 4 displays disrupted GO term and Reactome pathway enrichment for genes identified as differentially expressed at non-teratogenic concentrations, teratogenic concentrations, and those which overlapped both concentration ranges.

**FIGURE 5:**
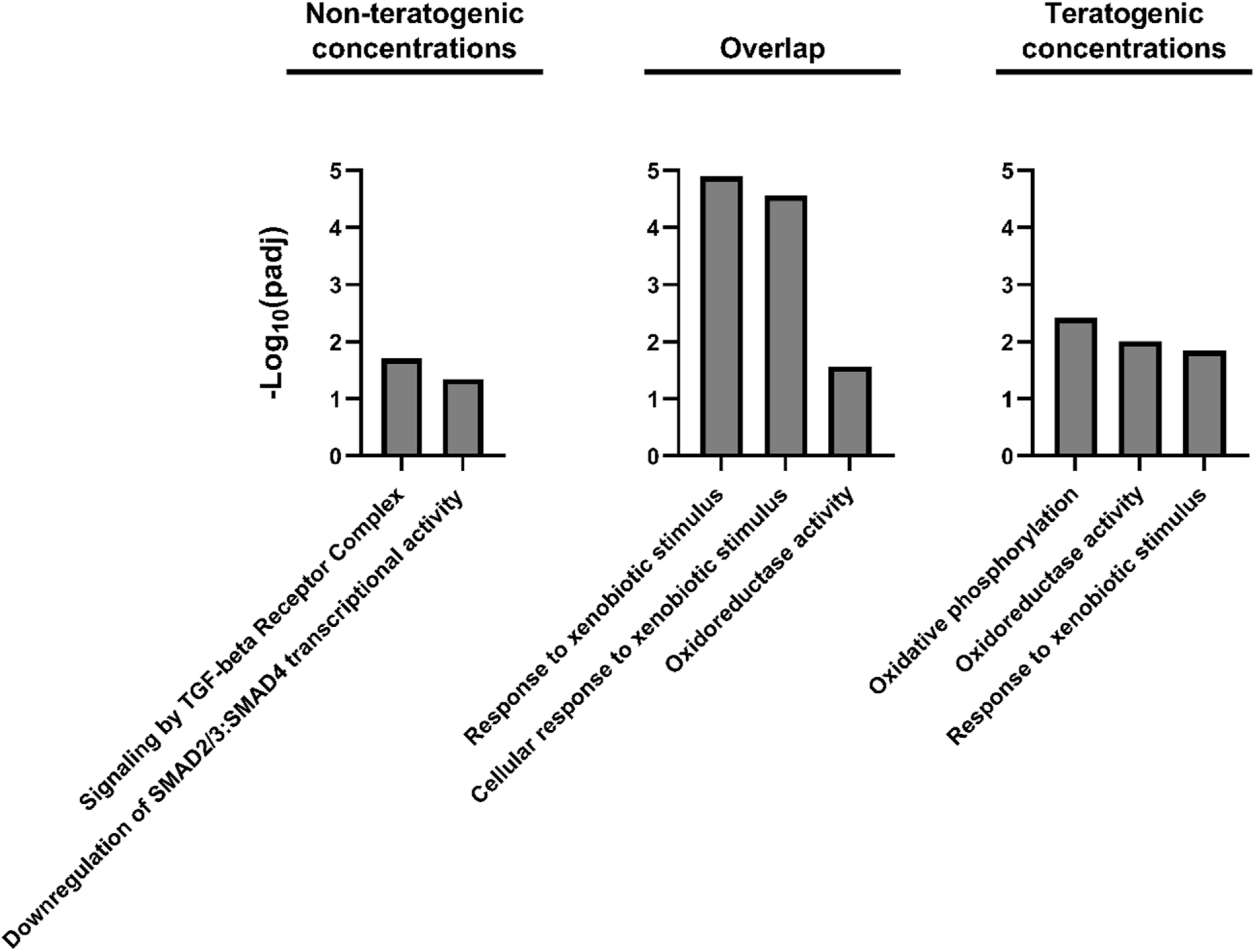
Gene Ontology and Reactome pathway enrichment for DEGs unique to non-teratogenic concentrations, DEGs overlapping at least one non-teratogenic and one teratogenic concentration, and DEGs unique to teratogenic concentrations.

350 genes were differentially expressed in at least one non-teratogenic and one teratogenic concentration (Figure 3B), allowing for analysis of expression of individual transcripts across concentrations. Figure 5 displays Log2FC values in response to increasing retene concentrations for these 350 genes. Genes were clustered using Ward clustering and several genes showed a strong concentration-response relationship with 19 genes (black and green clusters) showing the greatest increase in expression in response to increased retene concentration. Table 2 shows Log2FC across concentrations for black and green cluster genes from Figure 5.

**TABLE 2:** Black and green heatmap cluster genes which showed the strongest change in expression in response to increasing retene concentration. Log2 fold change values are shown for any gene with an adjusted *p* value <0.05. NA = adjusted *p*-value exceeded cutoff. Genes are ordered by the number of concentrations at which they showed significant differential expression. Functional roles were identified using AmiGO 2 (Ashburner et al., 2000; Carbon et al., 2009; Gene Ontology, 2021).

Gene	Heatmap cluster	Functional role	0.512 µM	1.28 µM	3.2 µM	8 µM	20 µM	50 µM
*cyp1a*	Green	Monooxygenase; biotransformation	2.92	4.62	6.22	7.44	8.44	9.57
*cyp1c1*	Green	Monooxygenase; biotransformation	NA	1.58	2.94	4.25	5.27	6.06
*sult6b1*	Black	Sulfotransferase; biotransformation	NA	0.45	0.76	1.38	1.69	2.51
*cyp1c2*	Green	Monooxygenase; biotransformation	NA	NA	2.06	3.08	4.26	5.31
*wfikkn1*	Black	Peptidase inhibition	NA	NA	2.46	3.64	3.74	4.26
*GeneID.110366352*	Black	Uncharacterized	NA	NA	1.03	1.65	2.37	3.21
*nfe2l2b*	Black	Transcription regulation	NA	NA	1.11	1.64	2.17	2.75
*ccn2b*	Black	Cell adhesion, heparin binding	NA	NA	0.68	1.32	1.81	2.10
*gstp1*	Black	Glutathione transferase; biotransformation	NA	NA	0.51	0.93	1.23	1.96
*cyb5a*	Black	Heme binding	NA	NA	0.52	0.81	1.2	1.8
*ahrra*	Green	Transcription regulation	NA	NA	NA	2.61	3.80	4.96
*fgf7*	Green	Cell proliferation	NA	NA	NA	1.96	3.26	4.48
*GeneID.103909114*	Black	Uncharacterized	NA	NA	NA	1.78	2.72	4.19
*GeneID.100536887*	Black	Uncharacterized	NA	NA	NA	1.55	1.92	3.10
*foxq1a*	Black	Transcription regulation	NA	NA	NA	1.22	1.92	2.65
*GeneID.108182865*	Black	Uncharacterized	NA	NA	NA	0.98	1.82	2.43
*mamdc2b*	Black	Uncharacterized	NA	NA	NA	1.35	1.76	2.26
*dhrs13l1*	Black	NADP-retinol dehydrogenase	NA	NA	NA	0.88	1.18	2.14
*per2*	Black	Circadian clock regulation	NA	NA	NA	1.03	1.37	1.77

**FIGURE 6:**
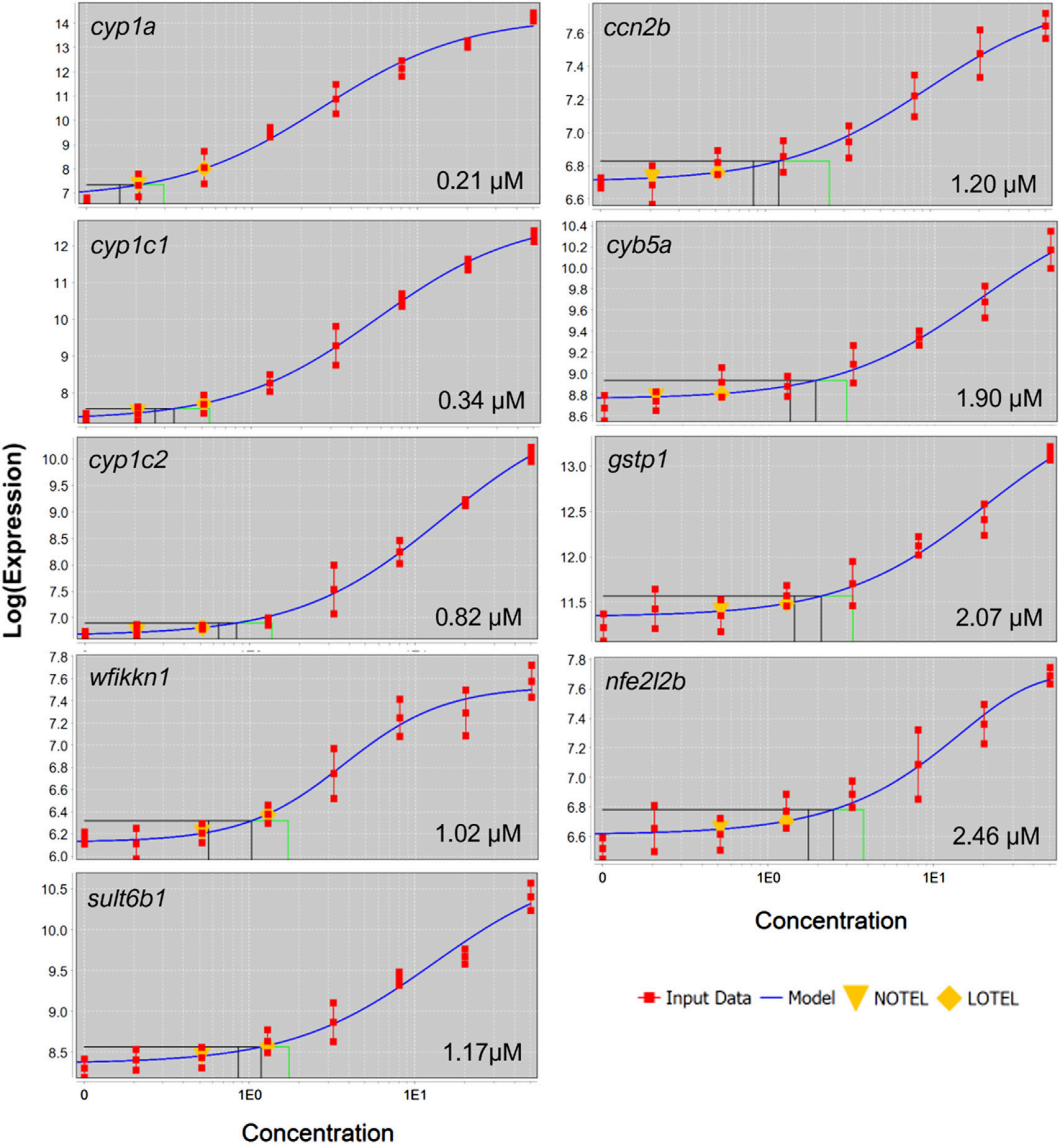
Concentration-response curves for black and green cluster genes which were differentially expressed in at four concentrations. Gene symbol and BMC are displayed in each panel.

BMDExpress2 was used to model concentration-dependent transcriptional changes for individual genes with significant concentration-response relationships. All genes which exhibited a response to increasing concentration were modeled and benchmark concentrations were identified. Concentration-response modeling revealed BMC values below 1 µM for several genes (Figure 6.) Each of these genes has been previously associated with AHR activators (Garcia et al., 2018; Shankar et al., 2021).

#### 3.2.2 Gene co-expression network

**FIGURE 7:**
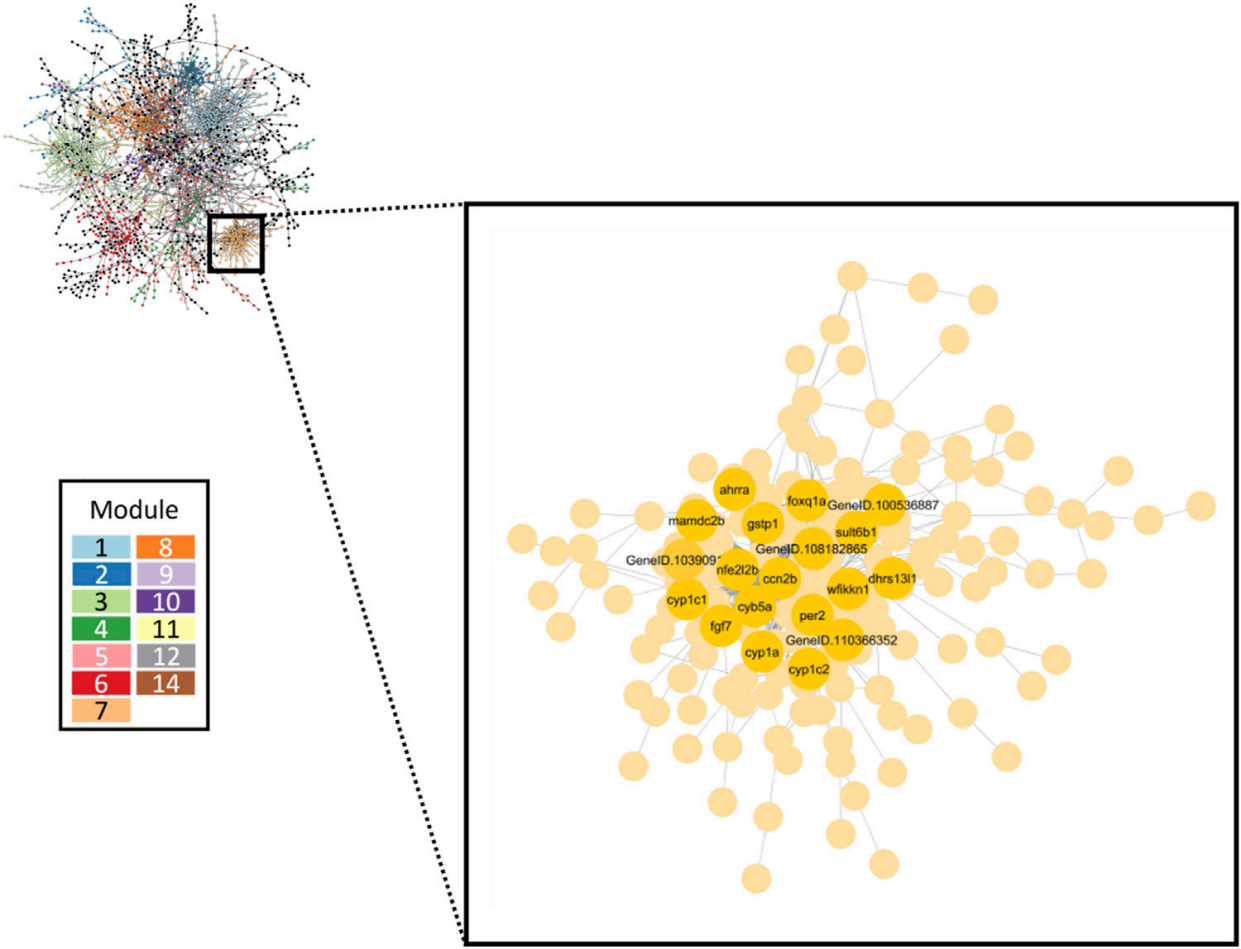
Gene co-expression network inferred from all retene concentrations. The largest 13 modules are differentiated by color. The inset box highlights Module seven which contained the greatest number of high responding genes. Black and green cluster genes which showed the strongest change in expression in response to increasing retene concentration are denoted.

A gene co-expression network was inferred by combining expression data from all 32 datasets using GENIE3 and were grouped into 30 separate modules ranging from 12 to 325 genes. Figure 7 displays the full retene network with the top 13 modules identified by node color. Overall, transcriptional responses to higher retene concentrations had an outsized effect on network structure (Supplementary Figure S1).

We conducted functional enrichment analysis on the largest 13 modules using g:Profiler. Eight modules were significantly enriched for at least one or more Gene Ontology (GO) terms (*p* < 0.05). Table 3 lists the top five modules of interest with their module size and GO term enrichment. Table 4 displays the top 20 genes with the highest degree (number of edges directly connected to a gene) and with the highest betweenness centrality (proxy for importance to network structure) from the full retene network. Many of the genes which showed the greatest response to retene had the highest degree in the network and were contained within Module 7.

**TABLE 3:** Gene network module ID, number of genes, and top GO terms of interest for significantly enriched modules (*p* < 0.05). Modules were selected based on size and significant functional enrichment.

Module	Size	Top GO terms of interest
3	279	HS-GAG biosynthesis; heparin metabolism; ion transport
4	128	Glutamate receptor activity; regulation of membrane potential
6	169	Apelin signaling pathway; ion channel activity
7	178	Response to xenobiotic stimulus, response to oxidative stress
8	240	response to wounding

**TABLE 4:** Top 20 genes with highest degree and betweenness in the full retene network. Functional roles were identified using AmiGO 2 (Ashburner et al., 2000; Carbon et al., 2009; Gene Ontology, 2021).

Gene	Functional role	Degree	Gene	Functional role	Betweenness
*ahrra*	Transcription regulation	40	*GeneID.110440109*	Uncharacterized	0.10
*cyp1c1*	Monooxygenase; biotransformation	35	*trpc2a*	Ion channel activity	0.09
*fgf7*	Cell proliferation	35	*spdya*	Protein kinase binding	0.08
*cyp1c2*	Monooxygenase; biotransformation	35	*ppig*	Isomerase activity	0.08
*cyp1a*	Monooxygenase; biotransformation	33	*sbno2a*	Chromatin DNA binding	0.08
*sult6b1*	Sulfotransferase; biotransformation	32	*phf20b*	Metal ion binding; histone acetylation	0.07
*tiparp*	Metal ion binding	30	*celf5b*	RNA binding	0.07
*GeneID.110366352*	Uncharacterized	30	*GeneID.100535592*	Uncharacterized	0.07
*wfikkn1*	Peptidase inhibition	30	*tmem54a*	Integral component of membrane	0.07
*GeneID.108182865*	Uncharacterized	29	*sik2a*	ATP binding	0.06
*nfe2l2b*	Transcription regulation	29	*cdc37*	Protein stabilization	0.06
*ccn2b*	Cell adhesion, heparin binding	29	*gsr*	Cell redox homeostasis	0.06
*lonrf1l*	Metal ion binding	27	*gap43*	Regulation of growth	0.06
*foxf2a*	Transcription regulation	27	*si.ch211.173n18.3*	Uncharacterized	0.06
*cdkn1ba*	Kinase inhibitor activity	26	*cers3a*	Sphingolipid metabolic process	0.06
*GeneID.103909114*	Uncharacterized	25	*josd1*	Protein deubiquitinase	0.06
*nfe2l2a*	Transcription regulation	23	*GeneID.103910736*	Uncharacterized	0.06
*cyb5a*	Heme binding	23	*gnai3*	GTP binding	0.06
*gstp1*	Glutathione transferase; biotransformation	22	*itpr1b*	Calcium channel activity	0.06
*per2*	Circadian clock regulation	21	*zgc.100868*	Endopeptidase activity	0.06

The network approach grouped genes based on similarity in expression and function and showed that Module seven was strongly linked to retene response (Figure 7) with all 19 of the black and green heatmap cluster genes central to this module. Module seven contained many known AHR-regulated genes and had 20.9% overlap with the AHR responsive module of the FRC/AHR network in Shankar, et al., 2021 (Shankar et al., 2021) (Supplementary Table S4). Functional enrichment analysis of Module seven revealed the most significant GO term was response to xenobiotic stimulus (Table 3), similar to the AHR responsive model from our previous publication. The remaining Module seven genes which were not common to the AHR responsive module from the previous study may be unique to the response to retene or may be additional AHR-responders that were uncovered through this study approach.

We next assessed the concentration-response relationship of Module seven gene expression with benchmark concentration modeling and network edge-weight analysis. First, we compiled BMC modeling data for all genes which comprise Module 7. The median BMC for Module seven genes was 5.23 µM (BMDL 2.67 µM; BMDU 9.59 µM), concordant with the BMC for teratogenicity of 5.5 µM. Next, edge weights were analyzed for all 24,758 possible edges that comprised Module 7. To assess which of these responded to increasing retene concentration, a ratio of edge weights was calculated for each Module seven edge in the original network compared to a network lacking each concentration. If the edge weight in the original network was greater than in the network lacking that concentration, we considered the edge responsive to that concentration. This was iteratively performed for each retene concentration for a total of seven ratios per edge. 1,152 edges had consistently higher weights in the original network and of these, 15 showed greater losses in edge weight as data from each higher retene concentration was removed, i.e., edge weight between the gene pairs comprising these edges became weaker as data from higher retene concentrations were removed. These genes were primarily low fold change genes (Log2FC < 1), though this suggested that co-expression of these particular gene pairs was strongly affected by retene (Supplementary Table S3).

## 4 Discussion

### 4.1 Observed phenotype is specific to early development

Exposure to retene during early development induced significant teratogenicity at concentrations 20 µM and above at 120 hpf. Teratogenicity was largely eliminated in AHR2-null fish, indicating the observed effects are dependent on AHR2. Previous studies have identified AHR2-mediated retene cardiotoxicity and cyp1a-induction patterns in early life-stage zebrafish (Scott et al., 2011; Shankar et al., 2019). This result and analysis of transcriptional response to retene support an AHR2-mediated toxicity pathway.

Preliminary data from our group, using the same exposure and screening paradigm presented here, showed that retene exposure beginning after 48 hpf did not induce developmental toxicity. These results indicate that the disrupted pathways which result in teratogenicity at 5 days post fertilization are disrupted prior to 48 hpf and gene expression changes observed at 48 hpf are likely representative of this disruption. Transcriptomic analysis at 48 hpf is an ideal timepoint in zebrafish as it precedes the onset of phenotype and the observed disrupted gene expression is likely to drive effects observed at 120 hpf (Haggard et al., 2018; Shankar et al., 2019; Dasgupta et al., 2021). In this study, zebrafish were developmentally exposed to retene and screened for early development-specific endpoints. We acknowledge that gene expression changes observed may be associated with non-developmental endpoints later in life such as disrupted reproduction, cognitive impairment and abnormal social behavior.

### 4.2 Concentration-response relationship of retene-induced transcriptional changes

#### 4.2.1 Differentially expressed genes increase with increasing concentration

At 5 days post fertilization, 20 and 50 µM retene exposures consistently induced teratogenicity not yet evident at 48 hpf, when transcriptomic analyses were performed. To assess the most sensitive phenotypically-anchored transcriptional events, we selected six concentrations with no observed toxicity and two concentrations with strong teratogenic effects at five to determine a concentration threshold at which phenotype-anchored DEGs were detectable. At the lowest concentration (0.205 µM), no DEGs were identified. At the next highest concentration, 0.512 µM, and above, DEGs generally increased with increasing retene concentration in both number and magnitude of expression. One concentration, 1.28 µM, did not follow this trend producing more DEGs than the next highest concentration. These were largely low fold-change genes (Log2FC < 1), though their altered expression may play a significant role in the cascade of transcriptional effects which lead to toxicity at higher concentrations.

At each retene concentration with a significant transcriptomic response, unique genes were observed. Across concentrations, patterns emerged of sets of genes differentially expressed at multiple concentrations with increasing or decreasing degree of change corresponding to increasing teratogenicity. Several individual genes showed a concentration-dependent increase in degree of differential expression. Black and green cluster genes from Figure 5 had a BMC ranging from 0.247 to 1.783 µM, indicating the concentration threshold which induced expression was well below that at which toxicity was observed. At the network level, the average BMC for all Module seven genes was 5.24 µM, similar to that of teratogenicity, 5.5 µM. Module seven consisted of many genes in the AHR2 pathway, meaning the threshold for significant induction of AHR2-associated genes was similar to the threshold for retene teratogenicity.

#### 4.2.2 Cyp1a is a biomarker of Aryl hydrocarbon receptor2 activation but not teratogenic outcome

Cytochrome P450s are a superfamily of enzymes which catalyze oxidation reactions in phase one biotransformation of xenobiotics (Hakkola et al., 1994; Carpenter et al., 1996; Lewis, 1996; Shimada et al., 1996). Increased expression of cytochrome p450 1a (CYP1A in zebrafish, or CYP1A1 in mammals) is a well-established biomarker of exposure to chemicals which activate the aryl hydrocarbon receptor pathway (Hu et al., 2007). In this study, elevated *cyp1a* was not associated with teratogenicity at very low concentrations, meaning that while its expression is considered a biomarker of AHR2 activation, it was too sensitive a response to serve as a biomarker of zebrafish teratogenicity later in development. The concentration-response transcriptional approach we have presented is useful for identifying transcriptomic biomarkers that are predictive of teratogenicity, though more work is needed to identify the DEGs which consistently and uniquely correlate with teratogenicity of PAHs. It is also possible that not only the elevated expression of a gene could serve as a predictive biomarker of teratogenicity, but a given magnitude of expression of specific genes could serve the same purpose, e.g. while elevated *cyp1a* does not predict teratogenicity, elevated *cyp1a* above a specific Log2FC may. By combining transcriptomic assessment across concentrations and network analyses for a larger number of PAHs, future studies could build upon this work to identify gene biomarkers or expression thresholds causally related to teratogenicity.

#### 4.2.3 Altered transforming growth factor-β signaling and oxidative stress may be responsible for retene teratogenicity

344 DEGs were unique to low, non-teratogenic, concentrations of retene. When assessed for functional enrichment, these genes were significantly enriched for Reactome pathway terms related to TGF-β signaling. TGF-β plays a fundamental role in growth and development and can inhibit expression and activity of CYP450 enzymes (Abdel-Razzak et al., 1994; Massague et al., 2000; Patterson and Padgett, 2000; Kubiczkova et al., 2012). Disruption of TGF-β signaling and increased TGF-β have been implicated in PAH-associated cardiac and respiratory toxicity (Curfs et al., 2005; Qin et al., 2018; Wang et al., 2021; Ma et al., 2022). AHR is critical for disruption of TGF-β in response to some PAHs, further supporting the AHR-dependence of transcriptional responses to retene, even when teratogenicity is not observed (Wang et al., 2021). In an adverse outcome pathway framework, altered TGF-β signaling may be an early key event in the retene toxicity pathway. Further investigation of the role of TGF-β in PAH-induced teratogenicity is warranted.

DEGs identified at higher, teratogenic concentrations, and those which spanned both non-teratogenic and teratogenic concentrations were significantly enriched for response to xenobiotic stimulus and ontology terms related to redox reactions and oxidative stress. Oxidative stress has been associated with teratogenicity and can play a role in PAH-induced toxicity (Ornoy, 2007; Lin et al., 2020). At higher retene concentrations associated with teratogenicity, disruption of redox balance appears to play a key role in toxicity response. This is consistent with previous studies implicating oxidative stress in retene-induced toxicity (Peixoto et al., 2019; Jiang et al., 2020). Whether effects of oxidative stress are responsible for retene-induced teratogenicity is still unclear, though the findings of this study support this hypothesis.

#### 4.2.4 Aryl hydrocarbon receptor-associated genes likely drive retene teratogenicity

While there is strong evidentiary support in the literature for which genes are induced by AHR activation, it is not clear if teratogenicity at high exposure concentrations of AHR activators is caused by 1) increased/decreased expression of direct AHR-regulated genes or 2) other cascading transcriptional responses which are only induced at concentrations at which teratogenicity is observed. The AHR battery contains a large number of genes, some of which have been studied significantly, while others have been only recently been identified and investigated (Timme-Laragy et al., 2007; Timme-Laragy et al., 2008; Planchart and Mattingly, 2010; Shankar et al., 2022). AHR activators can also induce a variety of toxic effects, not all of which involve the same AHR-regulated genes, highlighting the need to better understand which transcriptional changes can be causally associated with specific toxicity endpoints (Walisser et al., 2005; Watabe et al., 2010; Yoshioka et al., 2011; Wang et al., 2015; Zhang et al., 2016; Wright et al., 2017). One question this study looked to address was whether retene-induced teratogenicity likely resulted from the DEGs unique to teratogenic concentrations or from increased magnitude of expression of the more sensitive genes (identified at low concentrations). There were 880 DEGs uniquely associated with teratogenic concentrations (Figure 3B). When these genes were assessed for functional enrichment, the observed enriched GO and Reactome pathway terms were very similar to the terms enriched by DEGs which spanned both non-teratogenic and teratogenic concentrations, meaning that while many new DEGs arose when teratogenicity was observed, these DEGs were not related to novel functions. Additionally, the concentration thresholds for differential expression of Module seven genes and observed teratogenicity were highly correlated. Module seven contained mainly AHR-associated genes and genes which were differentially expressed at low, non-teratogenic retene concentrations. This high threshold correlation and lack of novel disrupted pathways associated with teratogenic concentrations support the hypothesis that DEGs identified at low, non-teratogenic, concentrations were responsible for retene teratogenicity, though their effects were only observed when their magnitude of change reached a certain threshold.

Results of this study suggest that early transcriptional response to retene is largely adaptive, with genes related to xenobiotic biotransformation (e.g. CYP450s) dominating the response. At teratogenic concentrations, many additional DEGs were induced, though they were related to the same molecular functions and pathways as their more sensitive counterparts. It is plausible that at low retene concentrations, the adaptive response was enough to withstand toxicity, but at higher concentrations, retene overwhelmed the adaptive response, leading to oxidative stress and, ultimately, teratogenicity at 5 days post-fertilization.

## 5 Conclusions and future directions

In this study, we definitively present a concentration-response relationship between increasing retene concentration and number of differentially expressed genes. Co-expression network analysis revealed one gene module significantly enriched for high retene-responders whose threshold for transcriptional response was similar to that for teratogenicity. DEGs were significantly enriched for TGF-β signaling at non-teratogenic concentrations and gene ontologies related to xenobiotic stimulus and redox balance at teratogenic concentrations, indicating altered TGF-β signaling may be an early response to retene and oxidative stress may play a key role in retene-induced teratogenicity at higher concentrations. We propose that at low retene concentrations, the pathway to teratogenicity was hindered by adaptive responses, including CYP450 metabolism, but at high concentrations, this adaptive response was overwhelmed and the pathway to teratogenicity proceeded. Finally, gene expression changes are often used as biomarkers of chemical toxicity, though, as we have displayed in this study, dysregulated gene expression does not always correlate with observed developmental toxicity in early life-stage zebrafish. Additional research using a larger number of diverse PAHs can deduce which gene expression changes are causally linked to teratogenicity *in vivo* and may uncover useful biomarkers of PAH-induced toxicity.

## Data Availability

The datasets presented in this study can be found in online repositories. The names of the repository/repositories and accession number(s) can be found below: https://www.ncbi.nlm.nih.gov/geo/, GSE203258.

## Supplementary material

Supplementary Table S1 Description of morphological endpoint binning.

Supplementary Table S2 All morphology data.

Supplementary Table S3 Edge weight shifts across retene concentration for Module 7 genes.

Supplementary Table S4 List of genes in Module 7 and the AHR-associated module from FRC/AHR network in Shankar, et al, 2021.
