# Supplementary material for: Concentration-response gene expression analysis in zebrafish reveals phenotypically-anchored transcriptional responses to retene
Source: Front Toxicol. 2022 Aug 25;4:950503. doi: 10.3389/ftox.2022.950503 (PMC9453431; doi:10.3389/ftox.2022.950503)
Supplement: Supplementary file 3 [file DataSheet1.PDF]

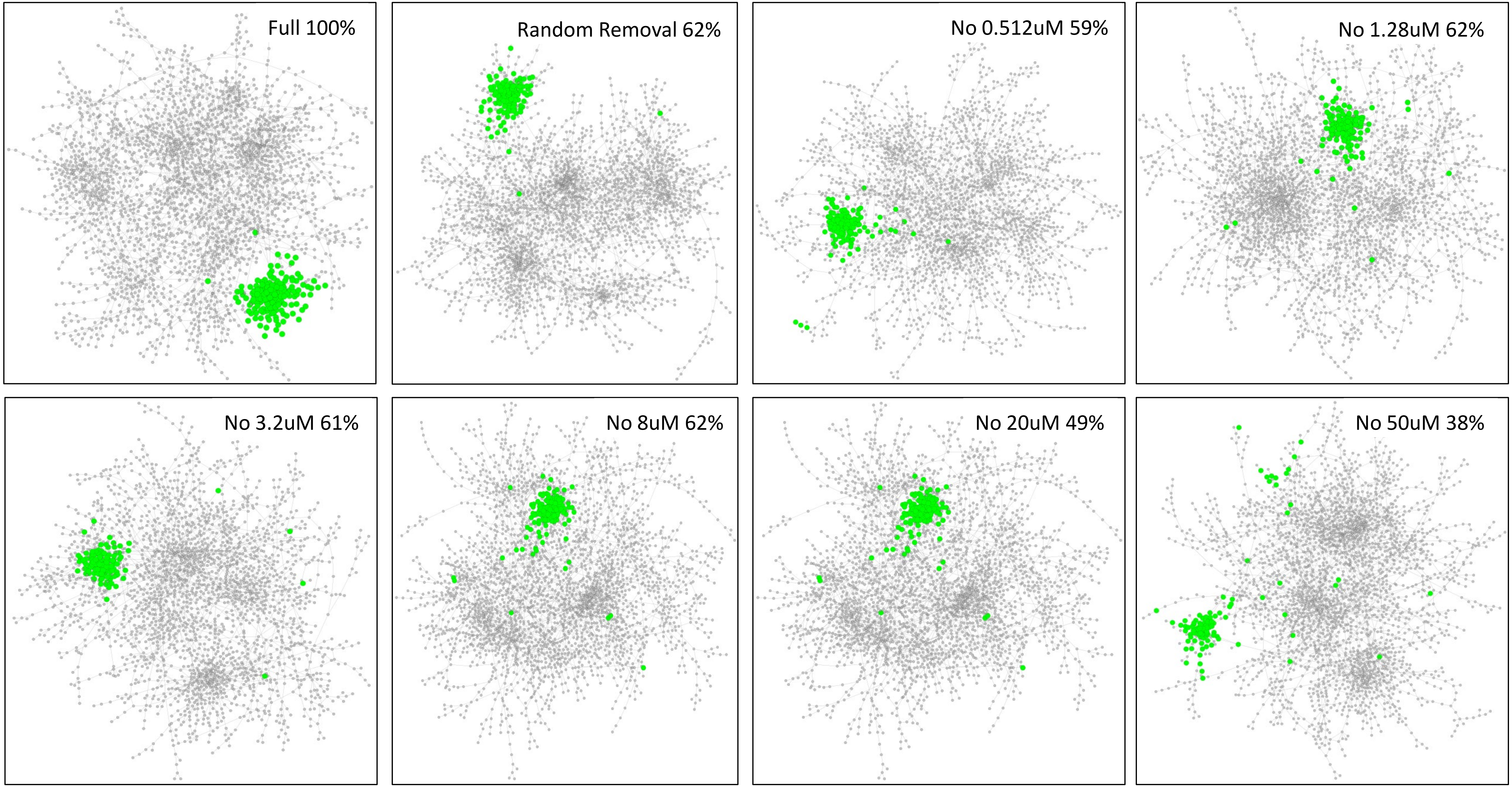

Figure S1. Retene module structure affected by data loss. A network was inferred with all data (Full 100%) with genes in the Retene responsive module (Module 7) colored green. Subsequent networks show the same Module 7 genes as the Module 7 is iteratively broken up through loss of data from samples treated with higher and higher concentrations of Retene. Which Retene concentrations that were removed are indicated (Random removal refers to an average network after removing random data) and the Jaccard overlap with the original module in the Full network is indicated.
